# Arsenic in Ascites

**Published:** 1844-08-10

**Authors:** 


ARSENIC IN ASCITES.

Dr. De Cavey gave arsenic in the dose of one twentieth of a grain twice a day, in a case of ascites following peritonitis, without any organic complication. The disease was of fifteen months standing. At the end of six weeks the abdomen was less tense, and was sensibly diminished in volume: the urine was at the same time increased in quantity. Uniform compression of the abdomen was then also employed, and at the end of six months there was scarcely any fluid remaining in the peritoneal cavity. The case is reported in the *Gazette Medicale.*

